# Platelet dense granule defect: experience in the French population

**DOI:** 10.1016/j.rpth.2026.103364

**Published:** 2026-01-29

**Authors:** Delphine Borgel, Agathe Beauvais, Cécile Bally, Manal Ibrahim-Kosta, Annabelle Dupont, Camille Paris, Caroline Vayne, Sophie Voisin, Valérie Goin, Guillaume Nam-Nguyen, Rémi Favier, Arnaud Dupuis, Sebastien Eymieux, Jean-Claude Bordet, Emmanuelle Blanchard, Claire Auditeau, Dominique Lasne, Annie Harroche, Marie-Christine Alessi, Mathieu Fiore

**Affiliations:** 1 Institut National de la Santé Et de la Recherche Médicale Unité Mixte de Recherche-S1176, Le Kremlin-Bicêtre, France; 2 Laboratoire d'Hématologie, Assistance Publique - Hopitaux de Paris, Hôpital Necker-Enfants malades, Paris, France; 3 Sorbonne Université, Assistance Publique - Hopitaux de Paris, Hôpital Saint Antoine, Paris, France; 4 Department of Hematology, Hemophilia Treatment Centre, Centre de Ressources et de Compétences des Maladies Hémorragiques Constitutionnelles, Hôpital Universitaire Necker-Enfants Malades, Paris, France; 5 Laboratory of Hematology, Centre de référence des pathologies plaquettaires, C2VN, Institut national de recherche pour l'agriculture, l'alimentation et l'environement, Institut National de la Santé Et de la Recherche Médicale, Aix Marseille Université, Marseille, France; 6 Service Hémostase et Transfusion, Centre Hospitalo-Universitaire Lille, Lille, France; 7 Service d'Hématologie-Hémostase, Centre Hospitalo-Universitaire de Tours, Institut National de la Santé Et de la Recherche Médicale Unité Mixte de Recherche 1327 ISCHEMIA, Université de Tours, Tours, France; 8 Laboratoire d’hématologie, Toulouse University Hospital, Toulouse, France; 9 Department of Hematology, Bordeaux University Hospital, Pessac, France; 10 Institut National de la Santé Et de la Recherche Médicale U1034, Biology of Cardiovascular Biology, Pessac, France; 11 French Reference Centre for Inherited Platelet Disorders, University Hospital of Bordeaux, Pessac, France; 12 Laboratoire d’hématologie, Assistance Publique - Hopitaux de Paris, Hôpital Armand Trousseau, Paris, France; 13 Institut National de la Santé Et de la Recherche Médicale Unité Mixte de Recherche_S1255, Strasbourg, France; 14 Etablissement Français du Sang-Grand Est, Strasbourg, France; 15 Microscopy Facility, US61 Analyse des Systèmes Biologiques, Tours University, Tours University Hospital, Tours, France; 16 Institut National de la Santé Et de la Recherche Médicale U1259 Morphogenèse et Antigénicité du VIH et des Virus des Hépatites, Tours University, Tours, France; 17 UR4609 Hémostase & Thrombose, Université Claude Bernard Lyon, Lyon, France

**Keywords:** bleeding assessment tool, blood platelets, platelet dense granules, platelet function tests, platelet storage pool deficiency

## Abstract

**Background:**

Platelet dense granule defect (DGD) is a frequent inherited disorder that is underdiagnosed due to its complexity and poor standardization of diagnostic tools.

**Objectives:**

To assess the prevalence of DGD in a large real-life French cohort of patients with an abnormal bleeding score.

**Methods:**

Patients with abnormal International Society on Thrombosis and Haemostasis Bleeding Assessment Tool scores, but no deficiency of coagulation or von Willebrand factor, and platelet counts > 100 × 10^9^/L, were recruited between December 2019 and March 2023 across 8 French expert centers. At the first visit, platelet function testing included light transmission aggregometry, whole-mount transmission electron microscopy, the mepacrine assay, and evaluation of CD63 expression. Patients with suspected DGD, based on prior testing, were included directly at the confirmatory visit (V2). DGD was defined by abnormal results on whole-mount transmission electron microscopy, mepacrine assay, or CD63 assay, and was considered confirmed if abnormalities were reproducible across both visits.

**Results:**

Of the 119 patients included at the inclusion visit, 66 had at least 1 abnormal dense granule test, including 19 with ≤2. Among the 54 patients seen at confirmatory visit , 40 had confirmed abnormalities, including 8 with at least 2 abnormal tests. Depending on diagnostic criteria, DGD prevalence ranged from 7.5% (≥2 abnormalities) to 37.4% (≥1 abnormality). Among 46 patients included at confirmatory visit, 30 had a confirmed DGD, including 11 with at least 2 abnormalities. No significant differences in age, sex, International Society on Thrombosis and Haemostasis Bleeding Assessment Tool scores, or bleeding history were observed between patients with or without DGD.

**Conclusion:**

DGD diagnosis in clinical practice depends on the criteria used. Standardized guidelines and repeated testing are essential for improving diagnostic accuracy.

## Essentials

• DGD is a frequent hereditary disease that is challenging to diagnose.
• Prevalence of DGD was evaluated in the French population.
• Prevalence of DGD ranged from 7.5% to 37.4% according to the diagnostic criteria used. ▪ Standardized guidelines and repeated testing are essential for improving diagnosis

## 1 Introduction

Platelet dense granule (DG) defect (DGD) is a heterogeneous subset of inherited platelet function disorders, characterized by the absence or reduced number of DGs, or by impaired storage of molecules critical for platelet activation and aggregation, such as adenosine diphosphate (ADP), adenosine triphosphate (ATP), calcium, or serotonin [1]. Indeed, DGs play a crucial role in amplifying platelet responses to vascular injury, and their dysfunction results in variable bleeding propensity, most commonly of a mucocutaneous type. However, whether DGD is associated with increased bleeding severity remains unclear. Several studies have reported that platelet function disorders are associated with abnormal bleeding scores, irrespective of the presence of DGD [[2], [3], [4], [5], [6]]. Others suggest that DGD could be among the most prevalent inherited platelet disorders in patients with a bleeding tendency [2,7,8]. The reported prevalence is high, ranging from 16% to 18% [2,7], highlighting the importance of improving detection strategies. Although major standardization efforts have been made recently, the diagnosis of DGD remains challenging [9]. Search for this disorder is recommended in first-line test for symptomatic patients, even in cases of normal platelet aggregation, and should include a δ-granule secretion marker [9]. Multiple tests are required to evaluate the content and function of DGs [10], including lumiaggregometry for ATP release, a flow cytometry (FCM)-based mepacrine assay, assessment of CD63 expression, whole-platelet serotonin and ATP/ADP content evaluation, and whole-mount platelet transmission electron microscopy (WM-PTEM) to assess granule numbers and content. However, none of these tests, by itself, provides sufficient specificity or sensitivity to confirm a diagnosis of DGD [11]. Consequently, a combination of tests is typically required across 2 independent visits, as recommended in the guidelines of the International Society on Thrombosis and Haemostasis (ISTH) Standardization Subcommittee (SSC) on Platelet Physiology [9]. However, there is no consensus on the minimum number or types of tests required to establish a diagnosis, reflecting the diagnostic difficulties posed by this disease. The latter are exacerbated in real-world clinical settings, as some tests, such as WM-PTEM or high-performance liquid chromatography (HPLC)-based quantification of granule content, require specialized equipment and expertise and are accessible only to a limited number of laboratories. Even in specialized centers, the situation is heterogeneous, as demonstrated by the results of the 2015 SSC/ISTH worldwide survey on platelet physiology [12]. Platelet nucleotides were measured in only half of the participating laboratories, and were performed as first-line assays in only 25% of them. Even though the situation has evolved over the past 10 years, with testing for DGD in first-line assays likely becoming more frequent, it remains heterogeneous across laboratories. In France, patients with platelet function disorders are mainly investigated in laboratories affiliated with the National Reference Centre for Inherited Platelet Disorders. This reference center is working to standardize practices in line with ISTH recommendations to improve patient care in France.

The current study aimed to assess the prevalence of DGD in a large, real-world French cohort of patients referred for bleeding evaluation with abnormal ISTH Bleeding Assessment Tool (BAT) scores. As secondary objectives, we also evaluated the performance and concordance of various diagnostic tools for identifying DGD.

## 2 Materials and Methods

### 2.1 Patients

Between December 2019 and March 2023, patients referred to the Reference Centers for Inherited Platelet Disorders of 8 French University Hospitals (2 in Paris and 1 each in Marseille, Bordeaux, Toulouse, Strasbourg, Lille, and Tours) for assessment of bleeding risk and exploration of hemostasis were prospectively included in the present study, “Diagnostic des Anomalies des GRAnules Denses plaquettaires dans les syndromes hémorragiques inexpliqués” (AGRAD). Inclusion criteria were as follows: (1) abnormal ISTH-BAT standardized scores [13] for men, women, and children (<18 years) ≥4, ≥6, and ≥3, respectively; (2) normal prothrombin time, activated partial thromboplastin time and/or coagulation factors levels (factor [F]II, FV, FVII, FVIII, FIX, FX, and FXI) ≥50%, and von Willebrand factor (VWF) activity > 50%; (3) platelet count > 100 × 10^9^/L and normal expression levels of platelet glycoprotein (GP) Ib and GPIIb.

Patients treated with drugs interfering with platelet function (evaluated in DGD exploration) were excluded, as well as patients with hematological malignancies, which can be associated with acquired DGD [14]. Moreover, none of the patients had received a platelet transfusion within 14 days before laboratory analysis.

The AGRAD study was approved by the local ethics committee (number 2019-53) and registered on ClinicalTrials.gov (NCT04095715). It was conducted in accordance with the Declaration of Helsinki. Patients were informed about the study, and those who declined to participate were excluded.

### 2.2 Study design

**Figure 1:**
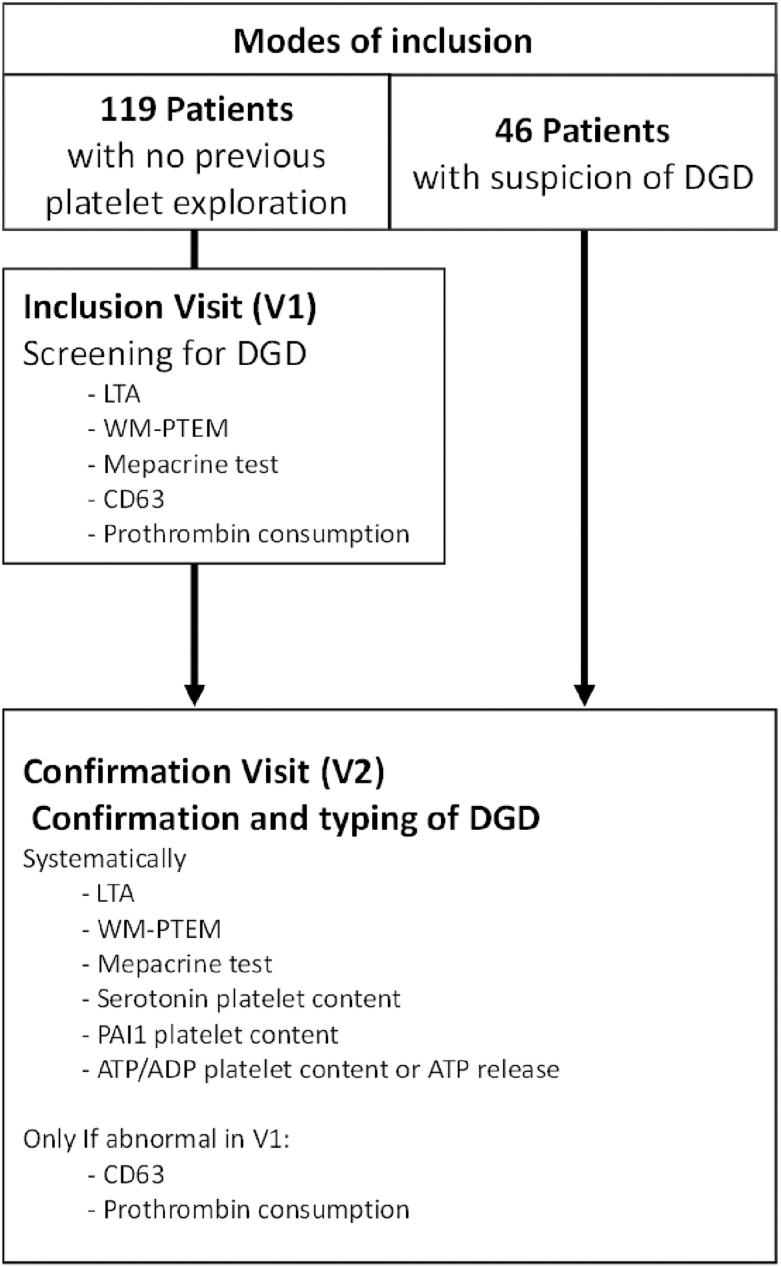
Study design. A total of 165 patients were included in the study. One hundred nineteen patients never had previous platelet exploration and underwent first-line platelet testing at the inclusion visit (V1). Forty-six patients already had an initial platelet exploration and were directly included in the confirmation visit (V2). Tests performed during V1 and V2 are indicated in the figure. ADP, adenosine diphosphate; ATP, adenosine triphosphate; DGD, dense granule defect; LTA, light transmission aggregometry; PAI-1, plasminogen activator inhibitor-1; WM-PTEM, whole-mount transmission electron microscopy.

The study design is depicted in Figure 1, showing the 2 possible modes of inclusion. Some patients underwent first-line platelet testing, including light transmission aggregometry (LTA), WM-PTEM, mepacrine test, and CD63 quantification at the inclusion visit (V1). A prothrombin consumption assay was also performed during V1 (Figure 1). Other patients had already benefited from an initial platelet exploration for a suspicion of DGD before the start of the study and were directly included in the confirmatory visit (V2). During V2, LTA, WM-PTEM, and mepacrine tests were systematically repeated, while CD63 quantification and prothrombin consumption were repeated only to confirm abnormal results from V1. Additional assays were performed, including quantification of intraplatelet serotonin, assessment of plasminogen activator inhibitor 1 (PAI-1) content, and quantification of either intraplatelet ADP/ATP content (expressed as a ratio) or ATP release after platelet activation.

### 2.3 Blood sampling and laboratory evaluation

Venous blood was collected in vacuum EDTA tubes for complete blood count. For hemostasis tests, blood was collected in tubes containing 3.2% sodium citrate at a 9:1 blood-to-citrate (v/v) ratio and processed within 4 hours. Platelet-poor plasma and platelet-rich plasma (PRP) were obtained by centrifugation of citrated blood for 10 minutes at 20 °C at 2500 × *g* or 200 × *g*, respectively, according to recommendations [15]. Routine clotting parameters, including prothrombin time, activated partial thromboplastin time, coagulation factors, and fibrinogen levels, were assessed using conventional methods available at the different hospital laboratories (Supplementary Methods). Complete blood count was performed on Sysmex XN analyzers.

Primary hemostasis was assessed by measuring VWF activity and platelet function, using methods implemented in each hospital laboratory according to recommendations [15]. The general principles of the methods used for platelet exploration are described below, and specific conditions for each laboratory are detailed in the Supplementary Methods.

LTA was performed using PRP, with platelet aggregation induced by different agonists, including ADP (5 μM), epinephrine (5 μM), arachidonic acid (1 mM), ristocetin (1.2-1.5 mg/mL), collagen (0.8-2 μg/mL), and thrombin receptor-activating peptide (TRAP; 10 μM), depending on the laboratory (Supplementary Methods). LTA tracings were analyzed by a hematologist, who provided an overall interpretation based on the percentage of maximum aggregation and the type of aggregation (reversible or irreversible).

Patients were also tested for platelet DGD by quantifying DGs using WM-PTEM. Platelet whole mounts were prepared after brief contact of formvar-coated grids with small drops of PRP, without the use of any staining reagent. Grids were then rapidly rinsed with distilled water, dried with the edge of a filter paper, air-dried, and sent for examination by transmission electron microscopy at 1 of the 3 equipped centers: IBiSA Electron Microscopy Facility, Tours University Hospital (JEOL-1011/1400 electron microscope; JEOL Ltd), CIQLE Lyon East University Hospital (JEOL JEM-1400 electron microscope), or Toulouse Rangueil Hospital (Hitachi HT-7700 electron microscope). DGs were identified and counted as previously described [16]. Mepacrine assays were performed by FCM at mepacrine concentrations of 1.1 to 2.4 μM before and after platelet activation with TRAP, thereby enabling assessment of mepacrine uptake and release. Mepacrine assays were considered abnormal if either uptake or release, or both, were outside the normal range defined by each participant’s laboratory. Finally, the expression of the platelet receptor CD63 was quantified by FCM on resting and TRAP-activated platelets.

Platelet nucleotide secretion was measured on a CHRONO-LOG aggregometer (CHRONO-LOG Corp.) after activation of PRP with different agonists (Supplementary Methods). Results were expressed as nmol secreted ATP/10^8^ platelets. Another option for whole-platelet nucleotide content evaluation was ADP and ATP by HPLC (Supplementary Methods). ADP and ATP quantities were calculated for each sample through a calibration curve with pure standard ADP and ATP. Results were expressed as an ATP/ADP ratio (normal range, 1.2-2.4).

Platelet serotonin content was quantified using either HPLC coupled with electrochemical detection of PRP lysates after thawing/freezing cycles, or through an ELISA (Supplementary Methods). Results were expressed as nmol/10^9^ platelets. Platelet PAI-1 content was quantified in serum by ELISA (Asserachrom PAI-1; Stago), since platelets are the unique source of PAI-1 in blood. Serum was incubated at 37 °C for 2 hours in a water bath, then centrifuged at 2300 × *g* for 10 minutes, and frozen at −80 °C for subsequent analysis. PAI-1 assays were centralized at La Timone Hospital in Marseille. Results were expressed as μg/10^9^ platelets.

Finally, a prothrombin consumption test was performed on whole blood collected in a glass tube (Z-tube, Vacutainer; Becton Dickinson). Prothrombin was measured in the serum obtained after whole-blood incubation for 4 hours at 37 °C, and residual prothrombin was expressed relative to plasma prothrombin. For each test, results were compared with each laboratory’s normal reference values by a hematologist, who classified them as normal or abnormal.

### 2.4 Statistical analysis

The distribution of each variable was tested for normality using the Shapiro–Wilk test. Normally distributed continuous data are expressed as mean ± SD, whereas skewed continuous variables are described as median and IQR. Categorical data are presented as numbers and percentages.

For multivariable analyses, we used Student’s *t*-test or Wilcoxon test for continuous variables according to their distribution, and the chi-squared or Fisher’s exact test for categorical variables, according to sample size. One-way analysis of variance or the Kruskal–Wallis test was used to compare >2 continuous variables, after checking for validity conditions.

All missing data were considered as not available and no imputation was performed. All analyses were 2-sided, and *p* values < .05 were considered statistically significant. Statistical analyses were performed using RStudio (Posit) and R version 4.1.2 (R Development Core Team, 2019).

## 3 Results

### 3.1 Patients and study design

**Figure 2:**
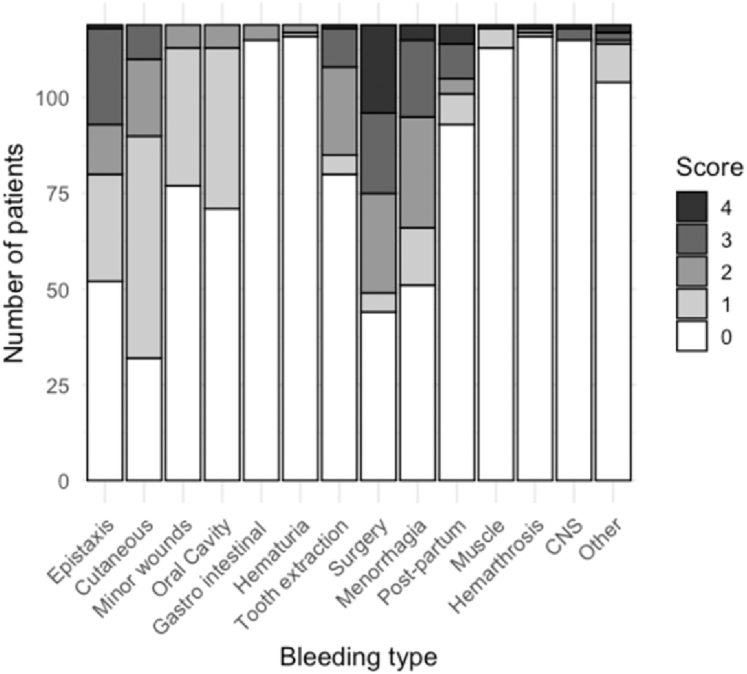
Details of the International Society on Thrombosis and Haemostasis Bleeding Assessment Tool score for patients included in the inclusion visit. Details of the International Society on Thrombosis and Haemostasis Bleeding Assessment Tool score for patients included in the inclusion visit, shown as the number of patients with reported bleeding symptoms by category and severity (4 indicates the most severe, 1 indicates the least severe). CNS, central nervous system.

A total of 119 patients with an abnormal ISTH-BAT score, comprising a majority of women (73.9%), were included in the AGRAD study at V1. The main characteristics, including demographic and clinical settings, as well as routine hemostasis tests for these patients, are presented in Table 1. Their mean age ± SD was 36.7 ± 19.5 years, and, according to inclusion criteria, none of these patients had coagulation factor or VWF deficiencies (mean VWF activity ± SD, 91% ± 32%). In addition, their mean platelet count ± SD was 257 ± 70 × 10^9^/L, and their platelets expressed normal levels of GPIb and GPIIb, excluding Glanzmann thrombasthenia or Bernard–Soulier disease. The median ISTH-BAT score was 7 (IQR, 6-9) across all patients, 7 (IQR, 5.5-8) in men (*n* = 19), 8 (IQR, 7-10) in women (*n* = 77), and 5 (IQR, 4-7) in children (*n* = 23). The most frequently reported symptoms were mucocutaneous bleeding (Figure 2), with menorrhagia in 77.3% of the women, cutaneous bleeding in 73.1% of the patients, epistaxis in 56.3%, and oral cavity bleeding in 40.3%. Interestingly, postsurgical bleeding was also frequent, reported by 68.9% of the patients. Finally, only 27.7% of the patients reported a family history of bleeding disorders, and 4 (3.4%) were syndromic (1 with Noonan syndrome carrying a mutation in the *PTPN11* gene, 1 with congenital disorder of glycosylation mutated in the *ALG13* gene, 1 Bruton agammaglobulinemia carrier with a mutation in the *BKT* gene, and 1 with cardiofacial-cutaneous syndrome of unknown etiology).

**Table 1:** Clinical and routine laboratory characteristics of the patients included in the inclusion visit.

Characteristics	*n*	Values
Age at inclusion (y)	119	36.7 ± 19.5
**Sex**		
Women		88 (73.9)
Men		31 (26.1)
Syndromic patients	119	4 (3.4)
Familial history of bleeding	119	33 (27.7)
**ISTH-BAT score**		
Abnormal total bleeding score	119	119 (100)
Median score (IQR)		7 (6-9)
**Routine laboratory main results**	**119**	
Platelet count (10^9^/L)	119	257 ± 70
PT (%)	114	100 ± 13
FII (%)	83	102 ± 18
FV (%)	74	106 ± 23
FVII (%)	69	93 ± 27
FX (%)	71	100 ± 21
aPTT (ratio)	158	1.05 ± 0.13
FVIII (%)	114	114 ± 39
FIX (%)	79	109 ± 21
FXI (%)	79	106 ± 26
Fibrinogen (g/L)	108	3.4 ± 0.79
VWF activity (%)	119	91 ± 32

Data are presented as *n* (%) or mean ± SD.

aPTT, activated partial thromboplastin time; F, factor; ISTH-BAT, International Society on Thrombosis and Haemostasis Bleeding Assessment Tool; PT, prothrombin time; VWF, von Willebrand factor.

**Figure 3:**
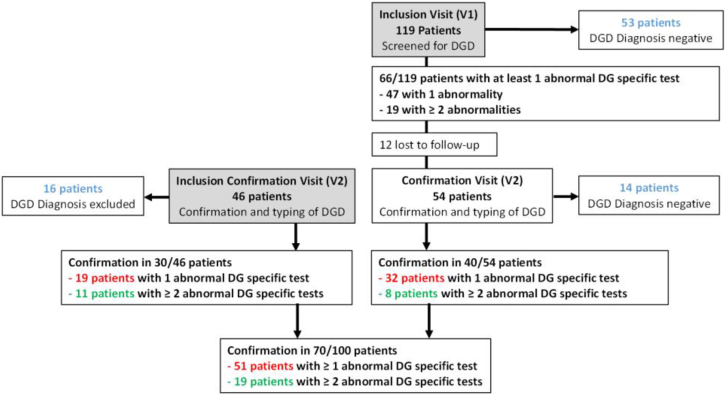
Flow chart of the study and identification of the patients with or without a dense granule defect (DGD). The 2 ways of inclusion in the study are indicated in the gray boxes. Dense granule (DG)-specific tests included whole-mount transmission electron microscopy, the mepacrine assay, and CD63 expression. DGD diagnosis is based on at least 1 abnormal DG-specific test. The DGD-pos-1 group corresponds to patients shown in red (70 patients). Patients with at least 2 abnormal specific tests belong to the DGD-pos-2 group and are shown in green (19 patients). Patients for whom the diagnosis of DGD has been excluded are shown in blue and belong to the DGD-neg group (83 patients). DGD-pos, platelet dense granule defect–positive; DGD-neg, platelet dense granule defect–negative.

### 3.2 Evaluation of platelet function and prevalence of DGD

The 119 patients underwent first-line platelet testing, with some receiving specific assessment of the presence or content of DGs (WM-PTEM, the mepacrine FCM assay, or CD63 quantification), while the other tests were not specific to platelet DGs but may be influenced by a DGD (LTA [6,7] and prothrombin consumption [17]).

The prevalence of DGD was assessed at V1. Sixty-six patients (55.5%) had an abnormality in at least 1 test specifically exploring DGs. Of these, 47 (DGD47 group) had only 1 abnormal test, whereas 19 (DGD19 group) showed abnormalities in at least 2 (Figure 3). Table 2 compares results in these 2 patient groups. The most frequent abnormality was WM-PTEM, for 72.7% and 83.3% of the DGD47 and DGD19 groups, respectively. In both groups, the median number of DGs/platelet was decreased compared with the normal count of ≥4 DGs/platelet. LTA for 1 or more agonists was more frequent in the DGD19 group (*P* = .03). Globally, particularly decreased responses were seen with TRAP (10 μM), collagen (0.8-2 μg/mL), and epinephrine. Abnormal responses to other agonists were less frequent and/or less frequently tested. A defect in the mepacrine FCM assay was observed in all but 1 patient in the DGD19 group, while this was the case for only a third of patients in the DGD47 group. A decrease in CD63 exposure after platelet activation was also observed, mainly in patients in the DGD19 group. Prothrombin consumption was more frequently abnormal in the DGD19 group (*P* = .004), yet with comparable mean residual prothrombin levels.

**Table 2:** Main results of platelet function testing in patients included in the inclusion visit.

Platelet function assay	*N*	Patients with 1 abnormality (*n* = 47) DGD47	*N*	Patients with ≥2 abnormalities (*n* = 19) DGD19
**WM-PTEM**	44		18	
Abnormal result		32 (72.7)		15 (83.3)
No. of DGs		2.9 (2.4-3.7)		3.1 (2.5-4.5)
Empty platelets (%)		26.5 (12-35)		20 (17-24)
**LTA**	47		19	
Abnormal result		15 (31.9)	19	9 (47.4)
(In at least 1 agonist)				
ADP 5 μM	43	7 (16.3)	15	1 (6.7)
Arachidonic acid 1 mM	47	4 (8.5)	19	2 (10.5)
TRAP 10 μM	46	10 (21.7)	19	7 (36.8)
Epinephrine 5 μM	29	5 (17.2)	8	1 (12.5)
Collagen 0.8-2 μg/mL	47	8 (17.0)	19	5 (26.3)
Ristocetin 1.2-1.5 mg/mL	47	1 (2.1)	19	0 (0)
**FCM**				
Mepacrine assay, abnormal result	42	13 (31)	19	18 (94.7)
CD63 expression, abnormal result	47	2 (4.3)	17	9 (52.9)
**Prothrombin consumption**	42		19	
Abnormal result		5 (11.9)		5 (26.3)

Data are presented as *n* (%) or median (IQR).

ADP, adenosine diphosphate; DG, dense granule; DGD, dense granule defect; FCM, flow cytometry; LTA, light transmission aggregometry; TRAP, thrombin receptor-activating peptide; WM-PTEM, whole-mount transmission electron microscopy.

As recommended by the SSC/ISTH on platelet physiology, the diagnosis of platelet secretion disorders must be established after confirmation by repeated laboratory testing [9]. The 66 patients were thus reconvened for confirmation at V2. Twelve were lost to follow-up (Figure 3), but a DGD with at least 1 abnormal test was confirmed in 40 of 54 patients tested; 8 had ≥2 abnormal tests (Figure 3). The prevalence was 8 of 107 (7.5%) when 2 abnormal tests were required to confirm a DGD diagnosis, and 40 of 107 (37.4%) when only 1 test was needed.

### 3.3 Test concordance between V1 and V2

For the 54 patients who underwent the confirmation V2, the concordance of results between V1 and V2 was systematically evaluated for each repeated test (Table 3). WM-PTEM was the most frequently discordant test (29.3% of the cases), followed by the mepacrine FCM assay (19%) and LTA (17.3%). Discordance in WM-PTEM resulted from both test normalization between V1 and V2 (31.3% of patients were abnormal at V1) and from abnormal WM-PTEM results in patients who were normal at V1 (22.2%). For LTA, results between V1 and V2 were discordant in 17.3% of patients; the highest concordance was observed with arachidonic acid and epinephrine as agonists (>90% of the cases). Discordance was more frequent with TRAP (10 μM), ADP (5 μM), and collagen (0.8-2 μg/mL), at 17.6%, 15.9%, and 14.0%, respectively. Normalization of the results between V1 and V2 was observed in half the patients with ADP and collagen.

**Table 3:** Concordance of tests between the inclusion and confirmatory visits.

Parameter	*n*	Normal V2 *n* (%)	Abnormal V2 *n* (%)	Total (%) discordance
**WM-PTEM**	41			**29.3**
Abnormal V1	32	10 (31.3)		
Normal V1	9		2 (22.2)	
**Mepacrine**	42			**19**
Abnormal V1	22	6 (14.3)		
Normal V1	20		2 (10.0)	
**LTA**	52			**17.3**
Abnormal V1	23	3 (13.0)		
Normal V1	29		6 (20.7)	
**ADP 5 μM**	**44**			**15.9**
Abnormal V1	6	3 (50.0)		
Normal V1	38		4 (10.5)	
**Arachidonic acid 1 mM**	**51**			**9.8**
Abnormal V1	5	1 (20.0)		
Normal V1	46		4 (8.7)	
**TRAP 10 μM**	**51**			**17.6**
Abnormal V1	16	3 (20.0)		
Normal V1	25		6 (17.1)	
**Epinephrine 5 μM**	**31**			**9.7**
Abnormal V1	7	1 (14.3)		
Normal V1	24		2 (8.3)	
**Collagen 0.8-2 μg/mL**	**50**			**14**
Abnormal V1	12	6 (50.0)		
Normal V1	38		1 (2.6)	

ADP, adenosine diphosphate; LTA, light transmission aggregometry; TRAP, thrombin receptor-activating peptide; V1, inclusion visit; V2, confirmatory visit; WM-PTEM, whole-mount transmission electron microscopy.

### 3.4 Type of DGD

During V2, additional platelet explorations, including assessment of serotonin levels and the ATP/ADP ratio, or ATP release upon platelet activation, were carried out to characterize the defect (Figure 1). A total of 100 patients were explored at V2: 54 patients described above for confirmation, and 46 patients who were directly included at V2 (Figure 2). A diagnosis of DGD was confirmed in 30 of the 46 patients based on at least 1 abnormal DG-specific test; 19 had only 1 abnormal test, and 11 had 2 or 3 abnormal tests. Interestingly, the ATP/ADP ratio and ATP release were abnormal in 29% and 38% of patients tested at V2, respectively, and a decreased platelet serotonin content was observed in 22.5% (Table 4). Abnormal mepacrine uptake was observed in 13.4% of patients, while abnormal release was present in 18.6% of the cases. Two patients with confirmed DGD had reduced platelet PAI-1 levels, indicating a combined defect in α- and δ-granules. Finally, among the 100 patients at V2, only 2 (2%) had a pure secretion defect with normal DG numbers (WM-PTEM) and content (serotonin, ATP/ADP, and mepacrine uptake), but an anomaly in at least 1 marker of DG secretion after TRAP activation (mepacrine release assay, ATP release, or CD63 exposure). For the 98 others (98%), DGD could be classified as a quantitative or granule content defect. This showed that an isolated platelet secretion defect was a very rare event in our population study.

**Table 4:** Results of complementary platelet function tests at the confirmatory visit.

Platelet function assay	*n*	Deficit, *n* (%)
**ATP/ADP ratio**	62	18 (29)
**ATP release**	50	19 (38)
**Serotonin content**	80	18 (22.5)
**PAI-1 content**	66	2 (3.0)

ADP, adenosine diphosphate; ATP, adenosine triphosphate; PAI-1, plasminogen activator inhibitor-1.

### 3.5 Comparison of patients with and without DGD

In the AGRAD study, DGD was confirmed in 70 of 100 patients (DGD-positive [pos]-1; Figure 3): 40 from V1/V2 and 30 from V2. Among these 70 patients, 19 had at least 2 abnormal tests (DGD-pos-2): 8 from V1/V2, and 11 from V2 (Figure 3 and Supplementary Table). DGD was therefore not retained (DGD-negative [neg]) in 83 patients: 53 with no abnormality affecting any of the 3 tests specifically exploring DGs after V1, and 30 (14 + 16) in whom DGD was not confirmed at V2 (Figure 3). These 3 groups (DGD-pos-1, DGD-pos-2, and DGD-neg) were compared and shown not to differ in terms of age, sex, ISTH-BAT score, familial history, or presence of syndromic clinical features (Table 5). There was also no difference in bleeding profile between the 3 groups of patients. As expected, disease-defining WM-PTEM, mepacrine and CD63 test were significantly more frequently abnormal in the DGD-pos-1/2 groups than in the DGD-neg group. A potential association was also investigated between DGD and the results of prothrombin consumption and LTA, not specifically designed for DG exploration. At variance from prothrombin consumption tests with no difference between patients with and without DGD (Table 5), abnormal LTA results were significantly more frequent in DGD-pos-2 patients than in controls, in particular for TRAP, collagen, and arachidonic acid agonists. Moreover, a significantly higher proportion of DGD-pos-2 patients had a reversible response to ADP compared with the DGD-neg group (*P* = .035). Of note, 1 of the 2 patients with reduced platelet PAI-1 levels and thus combined α- and δ-granule deficiency belonged to the DGD-pos-1 group, and the other to the DGD-pos-2 group.

**Table 5:** Comparison of patients with and without a dense granule defect.

Group	DGD-neg *n* (%)^a^	DGD-pos-1 *n* (%)^a^	DGD-pos-2 *n* (%)^a^	*P*
	*n* = 83	*n* = 70	*n* = 19	
**Age (y) at inclusion, mean ± SD**	38.2 ± 17.4	36.5 ± 20.4	38.8 ± 17.9	ns
**Gender**				ns
Female	66 (79.6)	49 (70.0)	14 (73.7)	
Male	17 (20.4)	21 (30.0)	5 (26.3)	
**Syndromic patients**	4 (4.80)	4 (5.80)	2 (10.5)	ns
**Familial history of bleeding**	26 (31.3)	22 (31.9)	2 (10.5)	ns
**ISTH-BAT score, % abnormal score (median; IQR)**				
Abnormal total bleeding score	100 (8; 6-9)	10 (8; 6-10.2)	100 (8; 6-10.5)	ns
Epistaxis	53.0 (1; 0-2)	55.1^b^ (1; 0-3)	52.6 (1; 0-3)	ns
Cutaneous	75.9 (1; 0-1)	66.7 (1; 0-2)	73.7^c^ (1; 0.5-2.5)	ns
Bleeding from minor wounds	39.8 (0; 0-1)	42.0 (0; 0-1)	63.2 (0; 0-1)	ns
Oral cavity	41.0 (0; 0-1)	42.0 (0; 0-1)	36.8 (0; 0-1)	ns
Gastrointestinal bleedings	2.4 (0; 0-0)	7.2 (0; 0-0)	10.5 (0; 0-0)	ns
Hematuria	1.2 (0; 0-0)	5.9 (0; 0-0)	5.3 (0; 0-0)	ns
Tooth extraction	34.9 (0; 0-2)	35.3 (0; 0-2)	36.8 (0; 0-1)	ns
Surgery	61.4 (2; 0-3)	63.8 (2; 0-4)	73.7 (3; 0.5-3.5)	ns
Menorrhagia	82.4 (2; 1-3)	66.7 (2; 0-3)	57.1 (1.5; 0-2)	ns
Postpartum hemorrhage	37.5 (0; 0-1)	29.2 (0; 0-1)	0 (0; 0-0)^c^	ns
Muscle hematomas	8.4 (0; 0-0)	5.8 (0; 0-0)	5.3 (0; 0-0)	ns
Hemarthrosis	1.2 (0; 0-0)	4.3 (0; 0-0)	5.3 (0; 0-0)	ns
CNS bleedings	3.6 (0; 0-0)	1.5 (0; 0-0)	0 (0; 0-0)	ns
Other bleedings	9.6 (0; 0-0)	11.8 (0; 0-0)	10.5 (0; 0-0)	ns
**WM-PTEM**				
Abnormal result	18 (25.0)	45 (80.5)^b^	11 (78.6)^c^	<.001^b^ and <.001^c^
**Mepacrine**				
Abnormal result	8 (14.8)	23 (59.0)^b^	9 (100)^c^	<.001^b^ and <.001^c^
**CD63**				
Abnormal result	5 (6.7)	13 (25.0)^b^	8 (61.5)^c^	.006^b^ and <.001^c^
**LTA**				
Abnormal result	28 (33.7)	33 (50.8)	12 (75)	ns and .002^c^
Abnormal results for the following agonists				
ADP 5 μM	12 (16.7)	10 (17.9)	4 (33.3)	ns
Arachidonic acid 1 mM	13 (15.7)	10 (15.6)	5 (31.2)^c^	ns and .044^c^
TRAP 10 μM	13 (16.7)	17 (30.9)	6 (50.0)^c^	ns and .006^c^
Epinephrine 5 μM	15 (32.0)	11 (26.2)	4 (44.4)	*Not analyzed*^d^
Collagen 0.8-2 μg/mL	17 (20.5)	22 (33.8)	8 (50.0)^c^	ns and .021^c^
Ristocetin 1.2-1.5 mg/mL	2 (2.7)	1 (1.6)	0 (0.00)	ns
**Prothrombin consumption**				
Abnormal result	7 (10.9)	10 (21.3)	2 (18.2)	ns

Biological results are those obtained during the inclusion visit. Percentages are indicated for available data.

ADP, adenosine diphosphate; CNS, central nervous system; DGD-pos, platelet dense granule defect–positive; DGD-neg, platelet dense granule defect–negative; ISTH-BAT, International Society on Thrombosis and Haemostasis Bleeding Assessment Tool; LTA, light transmission aggregometry; ns: nonsignificant; TRAP: thrombin receptor-activating peptide; WM-PTEM, whole-mount transmission electron microscopy.

a Unless otherwise specified.

b Significant difference between DGD-neg and DGD-pos-1 groups.

c Significant difference between DGD-neg and DGD-pos-2 groups.

d The variable was not analyzed because too much data were missing (>50% of missing data in at least 1 group).

## 4 Discussion

While the secretion of DGs is essential for platelet activation, many uncertainties remain regarding the hereditary defects of these intracellular organelles. These diseases appear to be a heterogeneous entity, both in terms of clinical and biological phenotypes, probably because of the complexity of DG biogenesis, which can be differentially affected [1].

DGD is difficult to diagnose because it requires a combination of tests, some of them being poorly standardized. The first objective of the AGRAD study was to evaluate the prevalence of platelet storage diseases affecting DGs in a French population with an abnormal hemorrhagic score. It has been reported that DGD is one of the most prevalent heritable platelet bleeding diseases [18]. Quiroga et al. [2] found a prevalence of primary secretion defects, as evidenced by decreased serotonin secretion, in 16.4% of 280 patients with mucocutaneous bleeding. Similarly, among patients with a bleeding tendency, 18.0% were diagnosed with a storage pool disorder based on a prolonged bleeding time and decreased platelet levels of total ADP and serotonin [7]. Guidelines for the diagnosis of inherited disorders of platelet function, including DGD, have been proposed by the SSC/ISTH on platelet physiology [9]. However, while these guidelines clearly state that platelet abnormalities should be confirmed at 2 separate visits, the number and type of abnormal tests used to characterize DGD are less clearly defined [9], and this may have an impact on the calculated prevalence of DGD. For example, in the AGRAD study, if it is considered that 1 confirmed abnormality affecting a DGD-specific test (WM-PTEM, mepacrine assay, or CD63 expression) is sufficient to diagnose DGD, the prevalence would be 37.4%, which is much higher than previously reported. If it is estimated that 2 confirmed abnormalities affecting DGs are required for the diagnosis of DGD, the prevalence in the AGRAD population is 7.5%, which is lower than previously reported prevalences. Thus, the choice of criterion for defining a DG anomaly is critical and has a major impact on the calculated prevalence.

The prevalence of DGD might have been underestimated in the AGRAD study as platelet secretion or content in nucleotides and serotonin was not assessed at V1, as recommended [19]. However, WM-PTEM, the mepacrine test (reported to have a high negative predictive value for DGD [20]), and CD63 expression assessment were performed as a screening tests for DGD. Indeed, the design of this real-world study was based on individual laboratory practice, with no harmonization of platelet function tests. Moreover, although platelet serotonin and ADP/ATP content, assessed by HPLC, are considered the new gold standard for the diagnosis of DGD [9], the cost is high and HPLC is not readily available as a first-line test. All the centers involved in this study belong to the MHEMO network, which hosts the French reference centers for platelet disorders. These expert centers are aware of the ISTH recommendations, and the major general statements proposed by the SSC/ISTH on platelet physiology were followed in the design of the study [9]. Moreover, recommendations about the diagnosis of inherited platelet function disorders specify that when secretion is defective, granule content should be measured as a second-step test [9,19], as was mostly performed in the present study. Surprisingly, we also observed a very low proportion of patients (2%) with pure platelet secretion defect in our study, but this might be related to the fact that investigations of DG content with second-line platelet assays were not performed in all patients.

The diagnosis of DGD has not yet been integrated in the practice of hemostasis laboratories. Indeed, it was previously reported, in a survey conducted by ISTH that more than half of laboratories do not use specific tools for DGD diagnosis [12]. Only 23.4% of laboratories used FCM, 21.3% measured ATP secretion using LTA, and only 5% used WM-PTEM [12]. Beyond the fact that tools for DGD diagnosis are not available in many laboratories, there is great variability in these tests. In this study, WM-PTEM showed the greatest variability, with discordant results between the 2 visits in almost 30% of cases. WM-PTEM was considered for a long time as the gold-standard test for DGD diagnosis. However, it has previously been reported to be variable, particularly in patients with a mild reduction in DG numbers [6], and is likely to overdiagnose DGD [21], even though standardization efforts have been made regarding preanalytical conditions, image analysis, and reference ranges [16]. WM-PTEM is now rather considered as a supportive test, while HPLC quantification of serotonin and nucleotide content tends to be the new gold standard for DGD diagnosis [9]. In this study, the reliability of serotonin content could not be evaluated, as these tests were performed only at V2. However, because they were centralized at 2 sites, variability was limited. The mepacrine test showed 19% discordant results, but efforts made for its standardization [22] could improve this otherwise easy-to-implement test in a laboratory familiar with FCM. Interestingly, a defect in the mepacrine test was rarely isolated in the AGRAD study. Discordant LTA results between the 2 visits (17.3%) were more frequent for TRAP (10 μM), ADP (5 μM), and collagen (0.8-2 μg/mL). This is consistent with previous reports showing that, under real-life conditions, normalization of LTA results in 20% of cases when the test was repeated [23] and high interindividual variability with low concentrations of activators [24]. However, highly standardized conditions have been reported to ensure LTA reproducibility in 90.4% to 93.3% of cases, with either abnormal or normal platelet aggregation [25]. Discrepancies between the results of tests carried out during 2 independent visits raised 2 questions: (1) should a normal result from the first visit be checked during a second independent visit, since both normalization and appearance of anomalies were observed at V2, and (2) should an unconfirmed result be checked a third time? Given the variability of the tests used to diagnose DGD, we believe that, in addition to confirming an abnormal result twice, as recommended by the latest guidelines, identifying DGD with at least 2 different tests would improve diagnostic accuracy.

LTA has been reported to be normal in 25% to 50% of patients with DGD [6,7,26], and, consistently, normal aggregation patterns were observed in many of the patients with DGD in the AGRAD study, regardless of the number of anomalies considered to define DGD. Interestingly, LTA in response to TRAP (10 μM), collagen (0.8-2 μM), and arachidonic acid (1 mM) were more frequently abnormal in patients with DGD, with 2 confirmed abnormalities, compared with those without DGD. A more frequent abnormal response to collagen and TRAP was expected because, at low concentrations, aggregation to these agonists depends on the secretion step [6,27]. Finally, no significant difference in terms of frequency in platelet response to ADP (5 μM) was observed in patients with DGD compared with those without DGD, as previously reported [6], even though the response to low doses of ADP (2.5-5 μM) was suggested to be sensitive to secretion [1,27], which probably explains the higher proportion of patients with a reversible response to 5 μM ADP in the group of patients with DGD and 2 confirmed abnormalities.

The association between prothrombin consumption and DGD was also assessed in this study. Indeed, it was recently reported that in a small series of patients with DGD, prothrombin consumption was lower in patients with abnormal ISTH-BAT scores [17]. Such an association could not be assessed in this study that enrolled patients with abnormal bleeding scores. However, the proportion of patients with abnormal prothrombin consumption was not significantly different between patients with and without DGD.

It was also questioned whether the intensity and profile of bleeding in patients with DGD were similar to those in patients without DGD. To this end, the ISTH-BAT score [19] was used to standardize the evaluation of bleeding, since it was recently shown to be a reliable tool for bleeding assessment in inherited disorders of platelet function [3]. No differences were observed in bleeding type or intensity between patients with or without DGD in the AGRAD study. This was expected since the study design, which focused only on patients with an abnormal hemorrhagic score, made it difficult to establish whether DGD leads to a higher hemorrhagic risk. Moreover, DGD failed to correlate with the ISTH-BAT score in many other studies [[2], [3], [4], [5], [6]].

This study has several limitations. It is a real-world study, reflecting the exploration of patients in several French centers with heterogeneity in their practice due to practical constraints. Although all the laboratories involved in the study followed SSC/ISTH recommendations [15], some discrepancies remain, particularly in the choice of agonists, commercial preparations, and, more importantly, agonist concentrations, a reported source of variability [24,28] compared with a single-center experience. In addition, some tests, such as platelet serotonin quantification or assessment of the ATP/ADP ratio or ATP release, were performed as second-line tests, and it cannot be ruled out that some patients were missed at V1, which could impact prevalence. Finally, the design of this study does not allow for recommendations to be made, as there is no internationally recognized gold-standard test for the diagnosis of DGD. A prospective study using standardized methods for the investigation of DGD is needed to improve current recommendations.

This study highlights the complexity and heterogeneity of diagnosing platelet DGD in routine clinical settings. The prevalence of DGD is highly dependent on the diagnostic criteria and testing modalities employed, with significant variability observed across methods and between visits, and identifying DGD using at least 2 different tests would improve diagnosis. Harmonized diagnostic strategies combining standardized first- and second-line assays, along with repeated assessments, and expert interpretation are necessary to ensure accurate identification of DGD. Establishing internationally accepted diagnostic criteria would be essential to improve patient care, facilitate epidemiological studies, and advance the understanding of platelet DGD.
