# Supplementary material for: Platelet dense granule defect: experience in the French population
Source: Res Pract Thromb Haemost. 2026 Jan 29;10(2):103364. doi: 10.1016/j.rpth.2026.103364 (PMC12966660; doi:10.1016/j.rpth.2026.103364)
Supplement: Supplementary Material [file mmc1.docx]

**Platelet dense granule defect: experience in the French population**

Delphine Borgel,^1^ Beauvais A,^2^ Bally C,^3^ Ibrahim-Kosta M,^4^ Dupont A,^5^ C Paris,^5^ Vayne C,^6^ Voisin S,^7^ Goin V,^8^ Nam-Nguyen G,^9^ Favier R,^9^ Dupuis A,^10^ Eymieux S,^11^ Bordet JC, ^12(^^[[1]](#footnote-1))^ Blanchard E,^11^ Auditeau C,^1^ Lasne D,^1^ Harroche A,^3^ Alessi MC,^4^ Fiore M.^8^

**Supplemental Methods**

**Laboratory evaluation, automated parameters**

*At Necker-Enfants Malades Hospital, Paris*

All automatized parameters were evaluated on ACL-TOP instruments (Werfen, Barcelona, Spain). Routine clotting parameters included prothrombin time (PT) (RecombiPlastin 2G, Werfen), activated partial thromboplastin time (aPTT) (C.K. Prest^®^, Stago, Asnières-sur-Seine, France), FII, V, VII and X (Factor deficient plasma, RecombiPlastin 2G^®^, Werfen), fibrinogen (thrombin reagent, Siemens, Munich, Germany), FVIII, IX and XI (Synthasil^®^, Factor deficient plasma, HemosIL^®^ Calibrator, Werfen), and VWF activity (HemosIL^®^ VWF Ristocetin Cofactor activity, Werfen).

*At La Timone Hospital, Marseilles*

All automatized parameters were evaluated on STA R MAX instruments (Stago). Routine clotting parameters included PT (STA-NeoPTimal^®^), aPTT (C.K. Prest^®^) FII, V, VII, and X (STA-NeoPTimal^®^, STA^®^-Deficient II, STA-Deficient V^®^, STA-Deficient VII^®^, and STA-Deficient X^®^), fibrinogen (STA^®^-Liquid Fib), FVIII, IX, and XI (C.K. Prest^®^, STA-Immuno Def VIII^®^, STA-Immuno Def IX^®^, and STA-Immuno Def XI). All reagents were from Stago. VWF activity was performed using HemosIL AcuStar Von Will RCo^®^ (Werfen).

*At Bordeaux University Hospital*

All automatized parameters were evaluated on ACL-TOP instruments (Werfen). Routine clotting parameters included PT (RecombiPlastin 2G^®^), aPTT (Synthasil^®^), FII, V, VII and X (RecombiPlastin 2G^®^, deficient plasma) fibrinogen *(*HemosIL^®^, Q.F.A Thrombin*),* FVIII, IX and XI (Synthasil^®^, deficient plasma, HemosIL^®^ Calibrator), and VWF activity (VWF:GPIbR Immunoturbidimétrie, HemosI^®^L). All reagents were from Werfen.

*At Toulouse Rangueil University Hospital*

All automatized parameters were evaluated on STA R Evolution instruments (Stago). Routine clotting parameters included PT (STA^®^-NeoPTimal), aPTT (C.K. Prest^®^), FII, V, X and VII (C.K. Prest^®^, STA-Deficient II, STA-Deficient V^®^, STA-Deficient VII^®^, and STA-Deficient X^®^), aPTT (C.K. Prest^®^), fibrinogen (thrombin reagent, STA^®^-Liquid Fib), FVIII, IX and XI (STA^®^-ImmunoDef VIII, STA^®^-ImmunoDef IX, STA^®^-ImmunoDef XI), STA^®^-Unicalibrator. All reagents were from Stago.

*At Paris Trousseau Hospital*

All automatized parameters were evaluated on STA R Max3 (Stago). Routine clotting parameters included PT (STA^®^-NeoPTimal), aPTT (STA^®^-PTT), FII, V, VII and X (STA-NeoPTimal^®^, STA^®^-Deficient II, STA-Deficient V^®^, STA-Deficient VII^®^, and STA-Deficient X^®^), fibrinogen (STA^®^-Liquid Fib), FVIII, IX and XI (STA^®^-ImmunoDef VIII, STA^®^-ImmunoDef IX, STA^®^-ImmunoDef XI). All reagents were from Stago except for VWF activity (Innovance^®^ vWF:Act, Siemens)

*At Strasbourg University Hospitals*

All automatized parameters were evaluated on a STA R Max (Stago) according to the manufacturer recommendation and using Stago reagents for clotting parameters including prothrombin time (STA-Neoptimal), activated partial thromboplastin time (STA-PTT Automate), fibrinogen (STA-Fib liquid), factor II, V, VII and X (STA-Deficient II, V, VII, X, and STA-Neoptimal,) and factor VIII (STA-Immunodef VIII and STA-CK Prest,). Willebrand factor antigen was evaluated with STA-Liatest VWF:Ag. Willebrand activity was evaluated with Innovance VWF Ac (Siemens) on STA R MAX.

*At Lille University Hospital*

Routine clotting parameters, including PT (Neoplastin R^®^), aPTT (Triniclot^®^ aPTT HS), FII, V, VII and X (Stago deficient plasma and Neoplastin R^®^), and fibrinogen (STA^®^-Liquid Fib) were performed on a STA R Evolution analyzer (Stago) with reagents from Stago, with some other reagents for FVIII, IX and XI (Siemens deficient plasma, Siemens, Saint-Denis, France, Triniclot^®^ aPTT HS, Stago). VWF activity (Innovance^®^ vWF:Act, Siemens) was assessed on a CS 2400 analyzer (Sysmex, Kobe, Japan).

*At Trousseau University Hospital, Tours*

All automatized parameters were evaluated on STA R MAX instruments (Stago), mostly with Stago reagents. Routine clotting parameters included PT (STA-NeoPTimal), aPTT (C.K. Prest^®^), FII, V (STA-NeoPTimal^®^, STA-Deficient II or V) fibrinogen (STA^®^-Liquid Fib, Stago), FVIII, IX and XI (C.K. Prest^®^). Siemens reagents were used for FVIII, FIX, FXI (deficient plasma), FVII and X (Thromborel^®^ S, FVII or FX-deficient plasma,) and VWF activity (GPIbM assay, Innovance^®^ VWF Ac).

**Platelet exploration**

*At Necker-Enfants Malades Hospital, Paris*

**Light transmission aggregometry** was performed using a thrombo-aggregometer (SD Medical, Th-v2-075, SD Innovation, Frouard, France). Platelet aggregation was induced, following manufacturer instructions, by different agonists, respectively ADP 5 μM (Chrono-PAR,^®^ CHRONO-LOG, Havertown, PA), arachidonic acid 1 mM (Stago), collagen 0.8 μg/mL (Chrono-PAR,^®^CHRONO-LOG), TRAP 10µM, epinephrine 5 µM (Stago) and ristocetin 1.2 mg/mL (Stago).

**Quantification of platelet receptor expression.** CD41a, CD42b, CD62P, and CD63 were evaluated by flow cytometry (FCM), on a FACS CANTO-II™ flow cytometer (BD Biosciences, Franklin Lakes, CA) , in whole blood, on resting platelets and after activation with TRAP-6 (Bachem, Bubendorf, Switzerland) 60µM, with the quantification kit Platelet GP Receptor^®^ (Biocytex, Stago), and CD63 PerCP-Cy™5.5 - Clone H5C6, (BDBiosciences).

**Mepacrine cytometry assay**. PRP was diluted to obtain a concentration of 10^7^ platelets/mL before incubation with 1.7 µM mepacrine (Sigma-Aldrich, St Louis, MO) or in the absence of mepacrine for 30 min at 37 °C. Then, platelets were stimulated or not with 60 µM TRAP-14 and analyzed on a FACS CANTO-II™ flow cytometer (BD Biosciences). An uptake ratio was calculated as the quotient of the Mean Florescence Intensity (MFI) of platelets incubated with or without mepacrine 1.7 µM. A release ratio was calculated as the MFI quotient of platelets incubated with mepacrine at rest divided by that of platelets incubated with mepacrine after stimulation by TRAP.

*At La Timone Hospital, Marseilles*

**Light transmission aggregometry** was performed using an APACT 4004 aggregometer (LABiTec, Ahrensburg, Germany). Platelet aggregation was induced by different agonists, respectively ADP 5 μM (Helena, Beaumont, TX), arachidonic acid 1 mM (Helena), collagen 2 μg/mL (Hyphen Biomed, Neuville sur Oise, France, Ref AG005K), TRAP-6 10 μM (Stago), epinephrine 5 μM (Helena), and ristocetin 1.25 mg/mL (Stago).

**Quantification of platelet receptor expression.** CD41 (CD41–PC5, Beckman-Coulter, Miami, FL), CD42b (CD42b–PC5, Beckman-Coulter), CD62P (CD62P–PC5, Beckman-Coulter), and CD63 (CD63–FITC, Beckman-Coulter) expression was evaluated by FCM on resting platelets (PRP) and after activation with TRAP-6 50 μM (Stago) and ADP 10 μM (Helena) on a Navios Cytometer (Beckman-Coulter).

**Mepacrine cytometry assay**: PRP was diluted to obtain 10^7^ platelets/mL before incubation with 1.1 or 2.4 µM mepacrine (Q 3251, Sigma-Aldrich) or in the absence of mepacrine for 30 min at 37 °C. Then, the platelets were stimulated or not with 23 μM TRAP-14 for 1 minute and immediately analyzed using a Navios Cytometer (Beckman-Coulter). The platelets mepacrine uptake was defined as the MFI ratio of platelets incubated with mepacrine to platelets incubated without mepacrine and the platelet mepacrine release was defined as the MFI ratio of resting platelets to stimulated platelets.

**The determination of intraplatelet serotonin concentration** was performed by a HPLC method (HPLC Dionex©, Thermo Scientifics, Waltham, MA) coupled with electrochemical detection (Precision Instruments©, Model 105, Marseilles, France). PRP samples were frozen at -80°C for at least 10 minutes and then thawed at 37°C in a water bath. This freeze-thaw cycle was repeated 3 times. After the final cycle, the samples were centrifuged at 12,000g for 2 minutes and frozen at -20°C for subsequent assay. Sample preparation and analysis were performed using the RECIPE Serotonin kit (HPLC ClinRep®, Recipe, Munich, Germany).

*At Bordeaux University Hospital*

**Light transmission aggregometry** was performed using ristocetin 1.2 mg/mL (Stago), ADP 10 µM (Calbiochem, San Diego, USA), arachidonic acid 1 mM (Nu Chek Prep, Elysian, MN, USA), TRAP-14 10 µM (Neosystem SA, Strasbourg, France), and collagen 2 μg/mL (Stago) in an APACT 4004 aggregometer (LABiTec).

**Quantification of platelet receptor expression.** CD41a and CD42b expression was measured in PRP using a calibrator kit (Biocytex, Marseilles, France). Platelet dense granules were also evaluated using 1.7 μM of **mepacrine** (Sigma-Aldrich) and CD63 antibody (granulophysin) (Beckman Coulter) before and after activation with TRAP 60 µM. Platelets were analyzed in a Cytomics FC500 or DxFlex flow cytometer (Beckman Coulter) and results were expressed as mean fluorescence intensity.

**Platelet ATP Release** was recorded in real time at 37°C with stirring on a dual-channel CHRONO-LOG aggregometer. The agonists used were ADP 10 μM (CHRONO-LOG), collagen 2 μg/mL (CHRONO-LOG), and TRAP-6 50 µM (Hart Biologicals Ltd, Hartlepool, United Kingdom). Platelet secretion was determined by measuring the release of adenosine triphosphate (ATP) using luciferin/ luciferase reagent (Kordia, Leiden, the Netherlands). Results were expressed as nmol of secreted ATP.

*At Toulouse Rangueil University Hospital*

**Light transmission aggregometry** was performed using a thrombo-aggregometer (Stago, TA 8-V3) using ADP 5 μM, arachidonic acid 1 mM (both from Sigma-Aldrich), collagen 0.8 μg/mL (Collagen Horm, Takeda, Tokyo, Japan), and ristocetin 1.2 mg/mL (Stago).

**Quantification of platelet receptor expression.** CD41a, CD42b, CD49s, and GPVI expression was evaluated with the quantification kit Platelet GP screen® (Biocytex, Stago). CD63 expression was also quantified before and after activation with different agonists (ADP 10 µM, TRAP 50 µM (Sigma-Aldrich), CRP 9 µg/ml (Collagen related peptide, Pplusmedical, Dublin, Ireland).

**Platelet ATP release** was assessed using the CHRONO-LOG lumiaggregometer after stimulation with ADP (10µM), collagen (3.3µg/mL), U46619 (5µM) or TRAP (25µM), all from Sigma-Aldrich.

**Mepacrine cytometry assay**. Diluted PRP was incubated with 2.0 µM mepacrine (Sigma-Aldrich) for 30 min at 37 °C. Mepacrine fluorescence was quantified before and after TRAP stimulation on a BD LSR II FORTESSA II (BD Biosciences).

*At Paris Trousseau Hospital*

**Light transmission aggregometry** was performed using a thrombo-aggregometer TA-8V (SD-Innovation). Platelet aggregation was induced, following manufacturer instructions, by different agonists, respectively ADP 5 μM (Sigma-Aldrich), arachidonic acid 1 mM (Sigma-Aldrich), collagen 1 μg/mL (Chrono-PAR,® CHRONO-LOG) and ristocetin 1.5 mg/mL (Helena).

**Quantification of platelet receptor expression.** CD41a, CD42b, CD62P, and CD63 expression was evaluated by FCM, on a NAVIOS™ flow cytometer (Beckman Coulter), on resting platelets and after activation with TRAP, with the quantification kit Platelet GP Receptor® (Biocytex, Stago).

**Mepacrine cytometry assay** was performed using Mepacrine Q3251-25G reagent (Sigma-Aldrich). Whole Blood was diluted 1:20 in Dulbecco’s Phosphate buffer Saline (D-PBS, Eurobio, Les Ulis, France). Subsequently, it was incubated with 1.4 μM mepacrine for 30 min at 37 °C. Mepacrine fluorescence was quantified before and after TRAP stimulation on a NAVIOS™ flow cytometer (Beckman Coulter)

*At Strasbourg University Hospitals*

**Light transmission aggregometry** was performed using an APACT 4004 aggregometer (LABiTec) using ADP 5µM (Ref 384, MAST Diagnostic, Bootle, UK), Collagen 1.25 µg/mL (Collagen Horm, Takeda), Ristocetin 1.25 (Helena) and Arachidonic Acid (SML1395, Sigma-Aldrich).

**Quantification of platelet receptor expression.** CD41a, CD42b, CD62P, and CD63 expression was assessed by FCM on a MACS Quant cytometer (Miltenyi Biotech, Bergisch Gladbach, Germany), on resting platelets and after activation with TRAP 50µM (PolyPeptide, Malmö, Sweden), with the quantification kit Platelet GP Receptor^®^ (Biocytex, Stago), and CD63 (PE labelled Clone H5C6, BD Biosciences). Data were analyzed using Kaluza Analysis software (Beckman Coulter).

**Platelet serotonin content** was measured in platelet pellets lysed by osmotic shock. Serotonin content was measured using the Serotonin ELISA Fast Track kit (LDN, Nordhorn, Germany) according to manufacturer recommendations. Quantification was performed using the SUNRISE spectrometer (TECAN, Männedorf, Switzerland).

**Platelet nucleotides quantification**. An aliquot of 450µL of platelet suspension was lysed by adding 50µL perchloric acid (6N, Merck, Darmstadt, Germany). After mixing and 10min incubation at 4°C, the suspension was centrifuged at 13,000 g for 5min at 4°C. The supernatant was mixed for 2min with trioctylamine/trichlorofluoromethane (v/v, Sigma-Aldrich), and centrifuged again. The 1260 High Pressure Liquid Chromatography (Agilent, Les Ulis, France) system was used to perform nucleotide (ADP and ATP) quantification. Briefly, 100µL of each sample were injected on an anion exchange column (PL1551-3802, Agilent) and eluted by a 0.5M phosphate buffer gradient (from 10 to 100%, 28min). Results were expressed as a ratio of ATP/ADP (Normal value 1.2 – 2.4)

*At Lille University Hospital*

**Light transmission aggregometry** was performed using a thrombo-aggregometer (TA-8V, SD Medical, SD Innovation). Platelet aggregation was induced, following manufacturer instructions, by different agonists: ADP 5 μM (Stago), arachidonic acid 1 mM (Stago), collagen 1.25 μg/mL (Collagen Horm, Takeda), and ristocetin 1.5 mg/mL (Stago).

**Quantification of platelet receptor expression.** CD41a, CD42b, CD62P, and CD63 expression was evaluated by FCM, on a DxFlex flow cytometer (Beckman Coulter), on resting platelets and after activation with TRAP (Biobyt, Cambridge, UK) 60µM, with the quantification kit Platelet GP Receptor^®^ (Biocytex, Stago).

**Platelet serotonin content** was measured in PRP samples by ultraperformance liquid chromatography on an HSST3 column (Waters, Milford, MA) with electrochemical detection (Thermo Scientific) after a single deproteinization step with perchloric and trichloroacetic acid.

**Platelet ATP release** was assessed using the CHRONO-LOG® lumiaggregometer after stimulation with TRAP 10 µM.

*At Trousseau University Hospital, Tours*

**Light transmission aggregometry** and ATP release assessment were performed using a CHRONO-LOG® lumiaggregometer (Chrono-log® 700). The different platelet agonists used were ristocetin 1.2 mg/mL (Helena), ADP 5µM (Helena), collagen 2 µg/mL (Collagen Horm, Takeda), arachidonic acid 1 mM (Helena), Epinephrine 5 µM (Helena) and TRAP-6 12.5 µM (Agrobio-Stago, Asnières-sur-Seine, France).

**Platelet ATP Release**: For ATP release measurement, a luciferin/luciferase reagent (Chrono-Lume®, CHRONOLO-LOG) was added 2 minutes before stimulation by ADP 5µM, collagen 5 µg/mL, arachidonic acid 1 mM, U46619 1 µM, epinephrine 5 µM, and TRAP-6 12.5 µM. According to the manufacturer's instructions, a pre-calibration of the lumiaggregometer was systematically performed using the Chrono-PAR ATP® standard reagent (CHRONOLO-LOG).

**Quantification of platelet receptor expression.** CD41a, CD42b, CD62P, and CD63 expression was assessed by FCM, on a BD Accuri™ C6 plus flow cytometer (BD Biosciences) using labelled antibodies (BD™ CD41a PerCP-Cy™5.5 - clone HIP8, BD™ CD42b FITC - clone HIP1, BD™ CD62P PE – clone AK-4, BD™ CD63 PerCP-Cy™5.5 - clone H5C6, BD Biosciences) on resting platelets and after activation with TRAP-6 50µM or ADP 10 µM.

**Supplemental Table 1:** Clinical characteristics and biological results of the 19 patients belonging to the DGD-pos-2 Group. Biological results are those obtained during V1 and V2 whenever available (indicated as follows: result V1/result V2) or performed only during V2 (ADP/ATP ratio, ATP release, serotonin and PAI1 content). Discordant results obtained between both visits are indicated (result V1/result V2).

**Supplemental Table 1:** Clinical characteristics and biological results of the 19 patients belonging to the DGD-pos-2 Group.

| **Patient identification** | **1** | **2** | **3** | **4** | **5** | **6** | **7** | **8** | **9** | **10** | **11** | **12** | **13** | **14** | **15** | **16** | **17** | **18** | **19** |
| --- | --- | --- | --- | --- | --- | --- | --- | --- | --- | --- | --- | --- | --- | --- | --- | --- | --- | --- | --- |
| Age at inclusion, years | 8.1 | 34.1 | 17.2 | 59.8 | 70.5 | 50.9 | 74.9 | 14.8 | 36.9 | 48.9 | 36.8 | 23.1 | 31.2 | 46.4 | 21.4 | 46.7 | 41.2 | 36.2 | 38.8 |
| Sex: Female (F) Male (M) | F | M | M | F | F | F | M | F | F | F | M | F | F | M | F | F | F | F | F |
| Syndromic patients (Yes/No) | Yes | No | No | No | No | No | No | No | No | No | No | No | No | **Yes** | No | No | No | No | No |
| Familial History of Bleeding (Yes/No) | No | No | No | No | No | No | No | No | No | No | No | No | No | **Yes** | No | No | **Yes** | No | No |
| **ISTH BAT score** |  |  |  |  |  |  |  |  |  |  |  |  |  |  |  |  |  |  |  |
| Total Score | 12 | 10 | 5 | 7 | 6 | 12 | 5 | 6 | 7 | 13 | 11 | 9 | 15 | 6 | 10 | 6 | 7 | 8 | 10 |
| Epistaxis | 3 | 3 | 2 | 0 | 0 | 3 | 0 | 2 | 0 | 3 | 3 | 0 | 3 | 0 | 1 | 0 | 0 | 0 | 4 |
| Cutaneous bleeding | 1 | 0 | 1 | 1 | 1 | 3 | 0 | 0 | 0 | 3 | 3 | 3 | 1 | 2 | 2 | 0 | 3 | 1 | 2 |
| Bleeding from minor wounds | 1 | 1 | 0 | 2 | 0 | 1 | 0 | 1 | 0 | 1 | 1 | 1 | 0 | 1 | 1 | 1 | 0 | 0 | 1 |
| Oral Cavity bleeding | 3 | 1 | 0 | 0 | 0 | 0 | 0 | 1 | 0 | 1 | 0 | 0 | 0 | 1 | 2 | 0 | 0 | 0 | 1 |
| Gastro intestinal bleeding | 0 | 0 | 0 | 0 | 0 | 1 | 0 | 0 | 0 | 0 | 0 | 0 | 0 | 0 | 0 | 0 | 3 | 0 | 0 |
| Hematuria, | 0 | 0 | 0 | 0 | 0 | 0 | 0 | 0 | 0 | 0 | 0 | 0 | 0 | 0 | 0 | 0 | 0 | 0 | 0 |
| Tooth extraction bleeding | 0 | 2 | 0 | 1 | 0 | 0 | 1 | 0 | 3 | 0 | 0 | 0 | 0 | 1 | 4 | 1 | 0 | 4 | 0 |
| Surgery bleeding | 4 | 3 | 2 | 3 | 3 | 4 | 4 | 0 | 3 | 0 | 4 | 2 | 4 | 1 | 0 | 0 | 1 | 3 | 0 |
| Menorrhagia, | 0 | 0 | 0 | 0 | 2 | 0 | 0 | 2 | 1 | 2 | 0 | 3 | 3 | 0 | 0 | 3 | 0 | 0 | 2 |
| Post-Partum hemorrhage | 0 | 0 | 0 | 0 | 0 | 0 | 0 | 0 | 0 | 0 | 0 | 0 | 0 | 0 | 0 | 0 | 0 | 0 | 0 |
| Muscle hematomas | 0 | 0 | 0 | 0 | 0 | 0 | 0 | 0 | 0 | 1 | 0 | 0 | 0 | 0 | 0 | 0 | 0 | 0 | 0 |
| Hemarthrosis | 0 | 0 | 0 | 0 | 0 | 0 | 0 | 0 | 0 | 2 | 0 | 0 | 0 | 0 | 0 | 0 | 0 | 0 | 0 |
| CNS bleedings | 0 | 0 | 0 | 0 | 0 | 0 | 0 | 0 | 0 | 0 | 0 | 0 | 0 | 0 | 0 | 0 | 0 | 0 | 0 |
| Other bleedings | 0 | 0 | 0 | 0 | 0 | 0 | 0 | 0 | 0 | 0 | 0 | 0 | 4 | 0 | 0 | 1 | 0 | 0 | 0 |
| **Routine Laboratory result** |  |  |  |  |  |  |  |  |  |  |  |  |  |  |  |  |  |  |  |
| Platelet count (10^9^/L) | 100 | 180 | 177 | 210 | 263 | 245 | 150 | 201 | 262 | 337 | 203 | 194 | 246 | 144 | 130 | 180 | 308 | 241 | 238 |
| **Results at V1/V2** |  |  |  |  |  |  |  |  |  |  |  |  |  |  |  |  |  |  |  |
| Abnormal LTA | **A**/**A** | N/N | **A**/**A** | N/N | *na*/N | *na*/**A** | *na*/N | N/N | **A**/**A** | N/N | **A**/**A** | **A**/**A** | **A**/N | **A**/**A** | **A**/**A** | **A**/**A** | **A**/**A** | **A**/**A** | **A**/**A** |
| For the following agonists |  |  |  |  |  |  |  |  |  |  |  |  |  |  |  |  |  |  |  |
| ADP 5 µM | N/N | N/N | N/N | N/N | *na*/N | *na*/N | *na*/N | *na/na* | *na/na* | N/N | N/**A** | N/N | N/N | **A**/**A** | **A**/N | **A**/**A** | *na*/N | *na*/N | **A**/**A** |
| Arachidonic Acid 1 mM | N/N | N/N | **A**/N | N/N | *na*/N | *na*/N | *na*/N | N/N | N/N | N/N | **A**/**A** | N/N | N/*na* | N/N | N/N | N/N | **A**/**A** | **A**/**A** | **A**/N |
| TRAP 6, low dose | *na*/**A** | N/N | **A**/**A** | N/N | *na*/N | *na*/**A** | *na*/N | N/N | **A**/**A** | N/N | **A**/**A** | **A**/**A** | **A**/N | N/N | *na*/N | N/N | *na*/**A** | *na*/**A** | **A**/**A** |
| Epinephrine 5 µM | N/N | N/N | N/N | N/N | *na/na* | *na/na* | *na*/N | *na/na* | *na/na* | *na/na* | *na/na* | *na/na* | *na/na* | N/N | **A**/**A** | **A**/**A** | *na*/**A** | **A**/**A** | **A**/**A** |
| Collagen Low dose | **A**/N | N | **A**/N | N | *na*/N | *na*/N | *na*/N | N/N | N/N | N/N | **A**/**A** | N/N | N/*na* | N/N | **A**/**A** | **A**/N | **A**/**A** | **A**/**A** | **A**/**A** |
| Ristocetin 1.2-1.5 mg/mL | N/N | N/N | N/N | N/N | *na*/N | *na*/N | *na*/N | N/N | N/N | N/N | N/N | N/N | N/na | N/N | N/**A** | N/*na* | N/N | N/*na* | N/*na* |
| **Tests specific for DGD** |  |  |  |  |  |  |  |  |  |  |  |  |  |  |  |  |  |  |  |
| CD63 | **A**/**A** | N/*na* | N/*na* | N/*na* | N/**A** | N/N | N/N | N/N | **A**/**A** | N/N | **A**/**A** | **A**/**A** | **A**/**A** | **A**/**A** | **A**/**A** | N/**A** | *na*/**A** | *na*/**A** | *na*/**A** |
| Mepacrin assay | *na*/N | **A**/**A** | *na*/**A** | **A**/**A** | *na*/**A** | *na*/**A** | *na*/**A** | **A**/**A** | **A**/**A** | **A**/**A** | **A**/**A** | **A**/**A** | **A**/**A** | *na/na* | *na*/**A** | **A**/*na* | *na/na* | *na/na* | *na/na* |
| Whole Mount Electronic Microscopy | **A**/**A** | **A**/**A** | **A**/**A** | **A**/**A** | *na*/**A** | *na*/**A** | *na*/**A** | **A**/**A** | **A**/*na* | **A**/**A** | **A**/*na* | N/N | N/*na* | **A/A** | N/*na* | **A**/**A** | *na*/**A** | *na*/**A** | **A**/**A** |
| Number of dense granules (median) | 2.3/2.3 | 0.4/3.0 | 2.4/3.6 | 2.7/1.5 | *na*/1.9 | *na*/1.4 | *na*/1.2 | 2.4/3.4 | 3.1/*na* | 1.8/1.9 | 2.2/2.2 | 6.9/6.8 | 4.5/na | 0.2/0.0 | *na/na* | 0.8/0.3 | *na/*0.0 | *na/*0.1 | 0.8/3.2 |
| ATP/ADP Ratio | **A** | N | N | **A** | **A** | *na* | **A** | N | **A** | N | **A** | *na* | *na* | *na* | *na* | **A** | **A** | *na* | *na* |
| ATP release | *na* | *na* | *na* | *na* | *na* | *na* | *na* | N | N | N | **A** | N | *na* | **A** | **A** | **A** | **A** | **A** | **A** |
| Serotonin content | **A** | N | N | N | **A** | **A** | **A** | N | N | **A** | **A** | *na* | *na* | *na* | *na* | **A** | **A** | **A** | **A** |
| **Prothrombin consumption** |  |  |  |  |  |  |  |  |  |  |  |  |  |  |  |  |  |  |  |
| Residual prothrombin (%) | N/N | N/*na* | *na*/N | N/N | *na*/N | *na*/N | *na/***A (13)** | N/*na* | N/*na* | **A(22)**/N | N/*na* | N/*na* | N/*na* | **A(14)/A(14)** | *na*/N | *na*/**A(29)** | *na*/N | N/*na* | *na*/**A (65)** |
| **Other tests** |  |  |  |  |  |  |  |  |  |  |  |  |  |  |  |  |  |  |  |
| **Platelet PAI1 content** | N | N | N | N | N | N | N | *na* | N | *na* | *na* | *na* | *na* | *na* | *na* | N | N | N | **A** |

*V: visit; LTA: light transmission aggregometry; N:Normal; A: Abnormal; na: not available*

1. ^()^ ✝This author has passed away since he participated in this study. [↑](#footnote-ref-1)
